# Implementing SMART Goals in a Radiology Research Mentorship Program: A Single-Institution Pilot Study

**DOI:** 10.1177/23821205261469924

**Published:** 2026-07-15

**Authors:** Syed Ibrahim, Thanh-Lan Bui, Sungmee Park, Shayan Saeed, Murad Aldoghmi, Parvaneh Hassani, Dillon Sommer, Justin Glavis-Bloom, Cameron Fateri, Daniel Kwan, David Floriolli, Edward Kuoy, Mohammad Helmy, Roozbeh Houshyar

**Affiliations:** 1 Irvine Department of Radiological Sciences, 12219 University of California, Orange, CA, USA

**Keywords:** SMART goals, mentorship, goal setting, medical education, research training, resident education, radiology education

## Abstract

**Background:**

Early engagement in research is essential for radiology trainees, yet many enter the field with limited experience and confidence. Structured goal setting—particularly through the SMART (Specific, Measurable, Attainable, Realistic, Time-bound) framework—may enhance research productivity and skill development. This study evaluated the impact of implementing SMART Goals within a radiology research mentorship program.

**Methods:**

Medical students and radiology residents participating in departmental research from August 2020–July 2021 completed SMART Goal worksheets and met individually with mentors to refine objectives, identify barriers, and create action plans. Scholarly output was compared descriptively with prior academic years (2016–2020). Participants completed pre- and post-intervention surveys assessing confidence across multiple research domains using a five-point Likert scale. Quantitative analyses included mean paired differences with corresponding 95% confidence intervals. Open-ended responses were thematically analyzed.

**Results:**

Nineteen of 22 invited trainees (86%) completed the SMART Goals process. Following implementation, participants demonstrated increased scholarly engagement compared with prior academic years, including participant-level credits for 45 accepted abstracts/conference presentations, 30 manuscripts/publications, and 7 successful grants. Confidence significantly improved across all evaluated domains, including scientific goal setting (mean increase 1.54), project initiation (1.05), manuscript preparation (1.21), and study design (0.62; all p ≤ 0.01). Qualitative feedback indicated that structured goal setting enhanced organization, accountability, mentor communication, and overall engagement.

**Discussion:**

Incorporating SMART Goals into research mentorship was associated with increased scholarly engagement and measurable gains in self-reported research confidence. Trainees reported that transforming broad intentions into actionable steps improved motivation, follow-through, and clarity in research planning. These findings align with adult learning principles and competency-based medical education models emphasizing autonomy, feedback, and self-directed growth.

**Conclusion:**

Formalized SMART Goal setting may be a feasible strategy to support research skill development, mentorship alignment, and scholarly engagement among radiology trainees. Broader, multicenter evaluations are warranted to assess long-term educational and career outcomes.

## Introduction

Clinical research remains fundamental to the creation of novel diagnostic and monitoring techniques, the development of innovative therapeutic approaches, and the continued advancement of medical specialties.^1^ Active research initiatives are particularly important in the field of radiology, as the evolution of imaging technologies and their effective translation into clinical practice drive both scientific progress and patient care forward.^2^ Therefore, a strong understanding of research methodologies and early engagement in investigative experiences are essential skills for radiologists and the next generation of medical scientists.

However, radiology has not always attracted research-oriented individuals, and there remains concern that too few radiologists are actively engaged in research, resulting in the scientific demands of the specialty not being fully met.^3-6^ To address this issue, prior literature has emphasized that the knowledge and practice of research should be introduced early in training to foster curiosity and a culture of inquiry among trainees.^2,7^ More recent radiology education literature has also emphasized that trainee research engagement depends not only on interest, but also on mentorship access, structured project development, protected research time, project feasibility, and clear expectations.^3-7^ Structured research tracks, resident-led initiatives, and departmental mentorship models have been described as ways to support scholarly productivity by giving trainees clearer pathways into research.^3-7^ However, many of these approaches require dedicated institutional infrastructure, sustained faculty involvement, or prolonged curricular time. In contrast, structured goal setting may provide a practical, low-resource method to strengthen existing research mentorship by helping trainees define project objectives, identify barriers, and maintain accountability over time.

A critical element in translating knowledge into practice is the deliberate creation of learning goals or action plans.^8,9^ Goals influence performance by directing focus, sustaining effort, and shaping strategies used to approach complex tasks.^9,10^ One widely accepted model for structuring such goals is the SMART (Specific, Measurable, Attainable, Realistic, Time-bound) framework.^11^ The SMART Goals framework is straightforward to teach and apply, easily retained, and, most importantly, produces actionable outcomes.^12-14^ Ideally, SMART Goals involve concrete steps that learners commit to implementing to refine their skills and attitudes, with an emphasis on measurable and meaningful results.

In research mentorship, trainees often begin with broad intentions, such as wanting to “do research,” “write a manuscript,” or “present at a conference,” but may lack a clear plan for translating those intentions into achievable steps. Without defined objectives, timelines, and mentor-aligned expectations, projects can stall despite trainee interest. The SMART Goals framework may help address this gap by converting broad research intentions into specific tasks, measurable milestones, realistic timelines, and shared expectations between trainees and mentors. In this way, SMART Goals may function not only as a goal-setting exercise, but also as a structure for mentorship, accountability, and project follow-through.

In this educational initiative, we implemented the SMART Goals framework within an existing radiology research mentorship program to provide trainees with a more structured approach to goal setting, project planning, and mentor communication. The purpose of this single-institution pilot study was to evaluate the feasibility and perceived educational impact of implementing SMART Goals among medical students and radiology residents participating in departmental research. Specifically, we examined trainee completion of the SMART Goals process, self-reported changes in research confidence, qualitative perceptions of the framework, and descriptive scholarly output compared with prior academic years.

## Methods

### Study Design

This was a single-institution pilot educational intervention evaluating the implementation of the SMART Goals framework within a radiology research mentorship program. The study used a pre- and post-program survey design to assess changes in trainee self-reported research confidence and a historical descriptive comparison of scholarly output from prior academic years to provide institutional context. The pre-SMART comparison group was not intended to serve as a randomized or contemporaneous control group.

### Participants

This study was reviewed and approved by the University of California, Irvine (UCI) Institutional Review Board (general IRB) (IRB #20416466); the requirement for informed consent was waived. Between August 1, 2020, and July 31, 2021, medical students and radiology residents participating in radiology research through the UCI.

Department of Radiological Sciences/CAR Lab research mentorship environment were invited to participate in the SMART Goals program. Participants were onboarded lab members conducting radiology research using departmental or institutional resources under principal investigator supervision. The intervention was implemented within an existing radiology research mentorship pipeline rather than as a general recruitment program for all trainees.

Participants included those who completed a SMART Goals worksheet and attended a formal review meeting to discuss their goals. Of the 22 invited trainees, 19 completed the SMART Goals worksheet and formal review meeting and were included in the final SMART Goals cohort. Three invited trainees did not complete the SMART Goals worksheet and/or formal review meeting and were not included in the final SMART Goals cohort. Formal reasons for non-completion were not collected.

For contextual comparison, research activity from prior academic years (August 2016–July 2020), before the introduction of SMART Goals, was reviewed descriptively to provide institutional background on trainee research engagement. The pre-SMART comparison group consisted of 28 medical students and radiology residents participating in the same departmental research mentorship environment under the same principal investigator mentorship before formal SMART Goal implementation. Because the pre-SMART period covered four academic years and the SMART Goals period covered one academic year, scholarly output was summarized descriptively and annualized when appropriate for comparison.

### Educational Intervention

The SMART Goals framework was introduced during an initial group meeting through a short lecture and supporting educational materials, including supplemental video content on SMART Goal development. Participants were guided through the process of formulating specific, measurable, and time-bound research goals related to ongoing or new projects. Participants then completed a SMART Goals worksheet focused on an ongoing or planned radiology research project.

After completing their worksheets, each participant met individually with the principal investigator and a research fellow to discuss goal feasibility, identify potential barriers, and outline an action plan. These dedicated individual review meetings lasted approximately 1 to 2 hours per participant and focused on feasibility, specificity, timeline, potential barriers, and alignment with available mentorship and project resources. These meetings emphasized mentorship, accountability, and goal refinement. Informal check-ins were conducted approximately monthly, with additional informal weekly check-ins available as needed. Formal progress reviews were also conducted at approximately 6 months and 1 year to reassess progress, barriers, and project direction.

The SMART framework was operationalized by applying each component to the trainee’s research project. “Specific” goals required trainees to define a clear project or scholarly output. “Measurable” goals required identifiable milestones, such as abstract submission, manuscript preparation, or grant-related progress. “Attainable” goals were reviewed with mentors for feasibility based on available time, project status, and resources. “Realistic” goals were aligned with the trainee’s research interests, radiology project goals, and career development. “Time-bound” goals included defined deadlines and were revisited during informal check-ins and formal 6-month and 1-year progress reviews.

To illustrate how the framework was applied to a clinical radiology research project, one de-identified SMART Goal stated: “I will publish a case series on uterine lipoleiomyoma by the end of March in order to hone my scientific writing skills and better prepare myself for a career as an academic radiologist.” In this example, the goal was specific because it identified a defined radiology project and scholarly product, measurable because completion could be tracked through manuscript preparation and submission, attainable because feasibility was reviewed with mentors, realistic because it aligned with the trainee’s academic radiology interests and available project resources, and time-bound because it included a defined completion deadline.

### Data Collection

Program evaluation included both objective and self-reported indicators of trainee engagement. Objective measures consisted of accepted abstracts/conference presentations, accepted manuscripts/publications, and successful grants produced during the academic year. For descriptive comparison, these outcomes were also reviewed relative to departmental output from prior years. Scholarly output was summarized using participant-level output credits. Under this approach, shared abstracts, manuscripts, or grants could contribute to more than one trainee’s total when multiple trainees contributed to the same scholarly product. This approach was used because the SMART Goals intervention was implemented at the individual trainee level, and trainees could contribute to both their own projects and collaborative projects involving other lab members.

To assess perceived educational impact, participants completed pre- and post-program surveys evaluating confidence in research-related skills. The survey instrument was developed de novo by the study team, was not adapted from a previously validated instrument, and was administered electronically using Microsoft Forms. The pre- and post-program surveys included 42 Likert-scale items across 11 research-related domains, including scientific goal setting, background research, study design and methodology, project initiation, data mining, data curation, data analysis, manuscript preparation, abstract and presentation preparation, research collaboration, and career development.

Items used a five-point Likert scale ranging from strongly disagree to strongly agree. The post-program survey also included four open-ended questions asking participants to reflect on what worked well, what could be improved, how orientation or reference materials could be presented more effectively, and whether setting a SMART Goal helped them with research. These survey responses allowed for both qualitative interpretation and quantitative evaluation of change over time, including differences ranging from 0.60 to 1.54 points across domains, each with associated p-values and 95% CIs.

### Data Analysis

Descriptive statistics were used to summarize participant demographics, scholarly output, and survey responses. Changes in confidence levels and research activity were examined to identify general trends in engagement and skill development. Productivity outcomes were interpreted descriptively because the pre-SMART group served as a historical comparator rather than a randomized or contemporaneous control group.

For survey comparisons, pre- and post-intervention means were analyzed using mean paired differences with corresponding p-values and 95% confidence intervals to quantify the degree of improvement across research skill domains.

Open-ended responses were reviewed for recurring themes that reflected the educational value of the SMART Goals framework, such as enhanced structure, accountability, mentor communication, and confidence in research participation.

## Results

### Participant Characteristics

Nineteen of the twenty-two invited trainees completed the SMART Goals worksheet (86% completion rate) and participated in a formal review meeting with the principal investigator and research fellow. This group included 15 medical students and 4 radiology residents, representing a range of training levels and research experience. The historical pre-SMART comparison group included 28 trainees, while the SMART Goals cohort included 19 trainees who completed the intervention. Participant characteristics are summarized in Table 1. Survey data were complete for all SMART Goals participants, allowing quantitative comparison of pre- and post-program scores across multiple research domains, as displayed in Table 2.

**Table 1.** Characteristics of Participants in the SMART Goals Educational Initiative

​	Pre-SMART goals cohort N (%)	SMART goals cohort N (%)
Total participants	28	19
Sex		
Male	21 (75)	17 (89)
Female	7 (25)	2 (11)
Level of training		
Resident	5 (18)	4 (21)
Medical Student	23 (82)	15 (79)
Highest level of education		
Undergraduate	19 (68)	12 (63)
Master’s	4 (14)	3 (16)
Medical Doctorate	5 (18)	4 (21)

**Table 2.** Survey Responses of the SMART Goals Group for Selected Research Domains

Research domain	Before SMART goal setting	95% CI	After SMART goal completion	95% CI	Difference (Δ)	*p*-value	95% CI
Scientific goal setting	2.57	1.69-3.45	4.11	3.47-4.74	1.54	<0.01	1.05-2.03
Study design/Research methodologies	3.38	2.66-4.10	4.00	3.41-4.59	0.62	<0.01	0.20-1.06
Project initiation	2.56	1.69-3.42	3.61	3.11-4.12	1.05	<0.01	0.63-1.56
Conference Presentations	3.59	2.73-4.45	4.19	3.69-4.70	0.60	0.01	0.15-1.06
Manuscript preparation	2.50	1.51-3.48	3.71	2.90-4.51	1.21	<0.01	0.62-1.80
Research for career development	3.32	2.70-3.93	4.25	3.69-4.80	0.93	<0.01	0.56-1.31

For each domain, the average participant ratings before they set their SMART goals and then at the end of the year after they had completed their SMART goal, the difference between the average ratings, and the *p*-value and associated 95% confidence intervals (CI) are shown.

### Scholarly Engagement and Clarity

Following the introduction of the SMART Goals framework, trainees demonstrated increased scholarly engagement compared with the pre-SMART comparison group. Scholarly output was summarized using participant-level output credits, meaning that shared abstracts, manuscripts, or grants could be counted for more than one trainee when multiple trainees contributed to the same scholarly product. The SMART Goals group generated 45 accepted abstract/conference presentation credits, 30 accepted manuscript/publication credits, and 7 successful grant credits among 19 trainees. In comparison, the pre-SMART group generated approximately 29 accepted abstract/conference presentation credits, 19 accepted manuscript/publication credits, and 1 to 2 successful grant credits among 28 trainees. These findings are summarized in Table 3. Conference outputs included oral presentations, poster presentations, and educational exhibits accepted at institutional, regional, national, and international meetings. To further address differences in project complexity, manuscript/publication outputs were manually classified by project type. In the pre-SMART group, classified outputs included 5 case reports, 11 original research projects, and 3 review articles. In the SMART Goals group, classified outputs included 13 case reports, 11 original research projects, 4 review or educational review articles, and 2 educational research/program evaluation projects. These categories demonstrate that scholarly output in both groups included a mix of project types with different levels of complexity, time commitment, and required baseline research experience, as shown in Table 4.

**Table 3.** Scholarly Output Before and After SMART Goals Implementation

Scholarly output	Pre-SMART group, n=28	Pre-SMART mean per trainee	SMART goals group, n=19	SMART goals mean per trainee
Accepted abstracts/conference presentations	29	1.04	45	2.37
Accepted manuscripts/publications	19	0.68	30	1.58
Successful grants	1–2	0.05	7	0.37

Counts reflect participant-level scholarly output credits. Shared abstracts, manuscripts, or grants could contribute to more than one trainee’s total when multiple trainees contributed to the same scholarly product. Pre-SMART values reflect a historical comparison group and were interpreted descriptively.

**Table 4.** Manuscript/Publication Output by Project Type

Manuscript/publication type	Pre-SMART group, n=28	SMART goals group, n=19
Case reports	5	13
Original research	11	11
Review articles/educational review articles	3	4
Educational research/program evaluation	0	2
Total classified manuscript/publication outputs	19	30

Manuscript/publication outputs were manually classified by project type to describe differences in scholarly product complexity. Individual projects were not weighted by time commitment, mentor involvement, difficulty, or required baseline research experience.

Quantitatively, trainees showed measurable growth in research confidence across all domains evaluated. Table 2 demonstrates that scientific goal-setting scores increased from 2.57 (CI 1.69–3.45) to 4.11 (CI 3.47–4.74), a mean improvement of 1.54 (p < 0.01). Project initiation increased by 1.05 points (CI 0.63–1.56), manuscript preparation improved by 1.21 points (p < 0.01), and study design improved by 0.62 points (CI 0.20–1.06These quantitative results support the observed educational improvements associated with the SMART Goals framework.

Qualitatively, trainees described that the SMART Goals structure helped them translate broad research intentions into actionable steps. Many reported feeling more organized and accountable to their timelines, and mentors observed improved consistency in follow-through and communication.

### Self-Reported Learning and Confidence

Survey results indicated that participants experienced meaningful growth in confidence across several domains of research skill development. The greatest improvements were reported in goal setting, project initiation, and manuscript preparation. Participants also noted gains in study design, abstract preparation, and understanding how research contributes to career development.

Narrative feedback reinforced these quantitative trends. Fourteen of the nineteen participants (74%) commented that SMART Goals positively shaped their research approach. Representative comments included:

“SMART Goals created a clear path toward achieving my objectives,”

“It helped me structure projects more wisely and stay accountable,” and

“It put me and my mentor on the same page, which made progress easier to track.”

These reflections suggest that structured goal setting supported participants’ sense of direction, confidence, organization, and collaboration within the mentorship process.

## Discussion

The implementation of individualized research goals through the SMART Goals framework was associated with increased engagement in research and improved self-reported confidence among medical students and radiology residents. Trainees who participated in the program demonstrated greater involvement in projects, conference presentations, and publications. Additionally, participants self-reported feeling more knowledgeable about and better able to set and achieve research goals, which is a skill they can continue to apply in the future. Furthermore, participants felt that setting SMART Goals positively influenced their motivation, organization, and communication with mentors, providing a structured approach to managing complex projects. These qualitative impressions were supported by measurable improvement across all research skill domains, each showing statistically significant increases with p-values ≤ 0.01 and positive mean differences, indicating consistent growth in confidence after the intervention.

Developing formal SMART Goals helped transform broad intentions of “doing research” into concrete, actionable steps. However, we do not interpret the worksheet alone as the active component of the intervention. Rather, the observed changes likely reflect the combined influence of structured goal setting, mentor-mediated refinement, feasibility review, barrier identification, roadmap creation, longitudinal accountability, and an existing research environment. This process encouraged accountability and ownership, which may have contributed to increased confidence and productivity. The most notable self-reported gains were in scientific goal setting, project initiation, and manuscript preparation, which are skills that are fundamental to independent research. These domains also showed the largest quantitative increases, including gains of 1.54 points in scientific goal setting and 1.21 points in manuscript preparation, both with supportive confidence intervals demonstrating improvement. Trainees described the framework as helping them visualize progress, anticipate barriers, and take greater initiative within their projects. Collectively, these observations suggest that the SMART Goals approach can serve as both an educational and motivational tool that supports early-career researchers in developing sustainable scholarly habits.

The SMART Goals framework aligns with several well-established principles in medical education, particularly goal-setting theory, adult learning theory, self-directed learning, and coaching-based approaches. Goal-setting theory emphasizes that specific and time-bound goals can direct attention, sustain effort, and clarify the strategies needed to complete complex tasks.^10^ In this project, those principles were operationalized by requiring trainees to define a clear research output, identify measurable milestones, assess feasibility with mentors, and establish deadlines for progress. The SMART framework is also consistent with prior literature showing that structured learning goals and individualized learning plans can support trainee development.^11-14^ Adult learning theory is relevant because trainees selected goals connected to their own research interests, career development, and ongoing projects.^15^ Similarly, self-directed learning and reflective practice were supported through mentor meetings, informal check-ins, and formal progress reviews, which gave trainees opportunities to reassess goals, barriers, and next steps.^16,17^ Finally, the mentor review process is consistent with coaching models in medical education, where feedback, accountability, and iterative reflection help learners develop competence over time.^18^ In this way, SMART Goals were not used only as a written exercise, but as a structure for aligning trainee autonomy with mentorship, feedback, and project follow-through.

The SMART Goals framework also aligns with competency-based medical education, particularly because it helps learners translate broad objectives into measurable outcomes. Within competency-based medical education, structured goals can help learners define progress, receive feedback, and connect individual development with observable outcomes.^19^ By supporting both scholarly growth and lifelong learning skills, the SMART Goals framework can bridge personal motivation with competency development.

Overall, the concept of formalized goal setting is something that has been considered useful in the guidance of trainees in research. For example, the National Institutes of Biomedical Imaging and Bioengineering promotes the myIDP (Individual Development Plan) tool, an online career-planning tool that PhD students and post-doctorates can use to reach short and long-term career goals.^20^ While similar, the SMART Goals framework appears to be more focused and therefore is potentially more suited to MD trainees, who typically have shorter dedicated time in which to conduct research. The quantitative improvements noted in our study, although modest in magnitude, suggest that even brief exposure to structured goal setting can enhance trainees’ readiness to engage in research and may serve as an accessible complement to other development tools such as myIDP.

Current literature has found that early involvement in research has the potential to increase the number of radiology trainees producing research and positively impacts trainees’ career development.^21-24^ Prior radiology literature has suggested that trainees who publish during residency may be substantially more likely to enter academic practice.^5^ Moreover, resident research experience has been shown to benefit residents in the form of opportunities to travel for conferences and competitiveness for fellowship applications.^5,25^ Our participants felt that the research they worked on in the SMART Goals framework helped them prepare for applying to the next step in their careers, including residency, fellowship, and future employment. Even for residents who do not end up pursuing research careers, engagement with mentors and senior colleagues at conferences is beneficial when searching for employment.^25^ Widespread application of deliberate goal setting can be considered an important skill to promote ongoing professional development for all radiologists.

Our experience implementing the SMART Goals framework in clinical radiology research reflects a single-institution initiative conducted over one academic year, which imposes several limitations. First, the small sample size limits statistical power and precision, and findings should be interpreted as exploratory and hypothesis-generating.

The survey instrument was developed de novo for this program evaluation and was not formally validated, which may limit comparability with other research training assessments. The relatively short timeframe limited our ability to assess the longer-term effects of the program, such as how participation influenced trainees’ future specialty choices or professional development. Future studies should explore the implementation of the SMART Goals framework across multiple institutions and over extended periods to evaluate its sustained educational and career impact.

Second, participants were already engaged in departmental radiology research, which may limit generalizability. Because the intervention was implemented within an existing research mentorship pipeline, participants may have had higher baseline motivation, mentorship access, and project availability than the broader trainee population. Additionally, the pre-SMART group was a historical comparator rather than a randomized or contemporaneous control group, limiting causal inference. Institutional conditions, project availability, trainee composition, and mentorship workflows may have changed over time, making it difficult to attribute differences in scholarly output to the SMART Goals intervention alone.

Furthermore, in our study design, we did not assign weights to the different projects undertaken as part of participants’ SMART Goals. Although manuscript/publication outputs were categorized by project type, individual projects were not weighted by complexity, time commitment, mentor involvement, or required baseline research experience. Case reports, review articles, original research projects, educational projects, abstracts, and grants require different levels of effort and cannot be interpreted as equivalent scholarly products. Future research should stratify projects by complexity, tabulate outputs by subtype and impact, and quantify the knowledge basis needed to begin projects to draw more in-depth conclusions.

Another limitation is that scholarly output was summarized using participant-level output credits. Shared abstracts, manuscripts, or grants could contribute to more than one trainee’s total when multiple trainees contributed to the same scholarly product. This approach reflects the individual-level nature of the SMART Goals intervention, but it may overestimate unique group-level scholarly products. Additionally, it was challenging to perform a comprehensive comparison of research experiences between the pre-SMART and SMART Goals groups due to the non-standardized nature of research experiences. Finally, due to the global COVID-19 pandemic, trainees in the SMART Goals group attended virtual meetings and collaborations, whereas in the years prior, research students and residents would often meet in person to discuss and review research projects. COVID-era virtual meetings may have influenced participation, mentorship access, and productivity independent of SMART Goals. This study did not assess what potential effects the change to online platforms might have had.

## Conclusion

In this single-institution pilot study, implementation of SMART Goals within a radiology research mentorship program was feasible and was associated with improved self-reported research confidence and greater observed scholarly engagement. Formalized goal setting may help trainees convert broad research intentions into structured, mentor-aligned action plans. Larger multicenter studies are needed to evaluate the long-term educational, scholarly, and career outcomes of SMART Goals-based research mentorship programs.

## Data Availability

The data that support the findings of this study are not publicly available because they include internal educational program evaluation and survey data. Data may be available from the corresponding author upon reasonable request, subject to applicable institutional and ethical approvals.*

## Appendix

### Abbreviations

SMART Goals: Specific, Measurable, Attainable, Realistic, Time-bound Goals
MD: Medical Doctorate
IRB: Institutional Review Board
myIDP: Individual Development Plan
COVID-19: Coronavirus Disease 2019
CBME: Competency Based Medical Education.
